# Host Innate Immune Responses of Ducks Infected with Newcastle Disease Viruses of Different Pathogenicities

**DOI:** 10.3389/fmicb.2015.01283

**Published:** 2015-11-17

**Authors:** Yinfeng Kang, Yanling Li, Runyu Yuan, Minsha Feng, Bin Xiang, Minhua Sun, Yaling Li, Peng Xie, Yangtong Tan, Tao Ren

**Affiliations:** ^1^College of Veterinary Medicine, South China Agricultural UniversityGuangzhou, China; ^2^Key Laboratory of Animal Vaccine Development, Ministry of AgricultureGuangzhou, China; ^3^Key Laboratory of Zoonosis Prevention and Control of Guangdong ProvinceGuangzhou, China; ^4^Key Laboratory for Repository and Application of Pathogenic Microbiology, Research Center for Pathogens Detection Technology of Emerging Infectious Diseases, Guangdong Provincial Center for Disease Control and PreventionGuangzhou, China; ^5^Guangdong Open Laboratory of Veterinary Public Health, Institute of Animal Health, Guangdong Academy of Agricultural SciencesGuangzhou, China

**Keywords:** host innate immune responses, duck, lung, thymus, Newcastle disease virus

## Abstract

Though previous studies have identified two strains of duck-origin Newcastle disease virus (NDV) with varying levels of pathogenicity, the relationship between the early-phase host innate immune response, and pathogenesis of ducks infected with these strains in the lungs and thymuses remains unclear. In this study, we compared the viral distribution and mRNA expression of immune-related genes in ducks following infection with two NDV strains, Duck/CH/GD/SS/10 (SS-10) and Duck/CH/GD/NH/10 (NH-10). Both NDV strains replicated systemically in tested tissues (i.e., small intestine, cecal tonsils, brain, lung, bursa of Fabricius, thymus, and spleen) and exhibited different biological properties in duck pathogenicity. Real-time quantitative polymerase chain reaction showed that the expression of *TLR3, TLR7, RIG-I, MDA5, IL-1*β, *IL-2, IL-6, IL-8, IFN-alpha, IFN-beta, IFN-gamma* in the lungs was significantly greater than in the respective thymus genes during the early post infection stage. However, in the lungs, the expression of *TLR3, TLR7, IL-1*β, *IL-2, IL-8, IFN-alpha, IFN-gamma*, and *MHC II* induced by SS-10 at 72 h post-inoculation (hpi) was less than with NH-10. Furthermore, the expression of *IL-6* and *IFN-beta* in the lungs and thymuses following infection with SS-10 was greater than that with NH-10 at 24 and 48 hpi. These results highlight important differences in host innate immune responses, courses of infection, and pathogenesis following NDV infection. Further studies should work to expand understandings of the molecular mechanisms related to NDV infection.

## Introduction

Newcastle disease (ND) is a devastating infectious disease in poultry. More than 241 bird species are reported to be susceptible to the disease’s causal agent, ND virus (NDV), thereby threatening severe economic burden in domestic poultry markets worldwide (Alexander, 2010). NDV is a negative-sense, non-segmented, single-stranded RNA virus that codes for six proteins—namely, nucleoprotein, matrix, fusion, hemagglutinin-neuraminidase, polymerase, and phosphoprotein—while the RNA editing of the phosphoprotein gene can additionally result in the expression of V and W (Alexander and Senne, 2008).

In general, ducks provide a natural reservoir for NDV, all genotypes of which— especially the Class I NDV— are perpetuated in the duck host, though ducks typically experience asymptomatic infection or little pathology for NDV lethal to chickens (Kim et al., 2007). Of the many NDVs isolated from domestic ducks in China in recent years, most strains are of low virulence, if not avirulent, though a high-virulence strain lethal to ducks has occasionally been isolated (Zhang et al., 2011; Kang et al., 2014). Nevertheless, little is known about host innate immune response to NDV in domestic ducks. Previous studies related to the topic have shown, however, that infection with NDV exhibiting a different level of virulence is associated with distinct cytokine expression patterns in the peripheral blood and spleen of chicken (Rue et al., 2011; Hu et al., 2012; Liu et al., 2012). By extension, modulation of cytokine responses may contribute to the pathogenesis of this highly virulent strain in chickens (Rue et al., 2011; Hu et al., 2012; Liu et al., 2012; Rasoli et al., 2014). Yet, no studies have focused on the host innate immune response of ducks infected with NDVs of different pathogenicities as a factor in determining the course of infection.

Pattern recognition receptors (PRRs) are responsible for identifying pathogen-associated molecular patterns that prompt the initiation and orchestration of innate and adaptive immune responses (Takeuchi and Akira, 2010). Toll-like receptors (*TLR 3/7/8/9*), as well as the retinoic-acid-inducible gene I (*RIG-I*), the melanoma differentiation associated gene 5 (*MDA5*), and the laboratory of genetics and physiology 2 (*LGP2*), have been distinguished as crucial sensors that recognize viral nucleic acids of RNA viruses, subsequently result in the production of interferon (IFN) types I and II, inflammatory cytokines, and chemokines, as well as the establishment of antiviral immunity (Bowie and Unterholzner, 2008; Takeuchi and Akira, 2010). Recent studies have demonstrated that the molecular basis for pathogenicity of NDV in chickens is determined by multiple proteins of the viral genome (Panda et al., 2004; Rout and Samal, 2008; Khattar et al., 2009; Samal et al., 2012, 2013; Mirza and Iorio, 2013). At the same time, other research has shown that the V proteins of NDV are responsible for blocking the antiviral functions of IFN, are associated with RIG-I and MDA5 and block the ‘downstream’ signaling pathway, suggesting that the detection of a strong host proinflammatory or antiviral response to NDV infection does not necessarily imply effective virus control (Huang et al., 2003; Andrejeva et al., 2004). Nonetheless, the roles of PRRs and proinflammatory cytokines in disease severity and outcomes in ducks infected systemically with NDV remain largely unknown.

Pro-inflammatory and anti-viral cytokines, chemokines, and IFN types I and II are involved in the early-phase host innate immune response of chickens following NDV infection. Many genes associated with an early-phase innate host response in spleens of infected chickens, such as *IL-6, MIP-3a, MDA-5*, and *IFIT-5*, were induced by California/S0212676/2002 (NDV-CA02), but not the lentogenic LaSota virus, at 1 day post inoculation (dpi) (Rue et al., 2011). The mRNA expression levels of *CXCLi2, IFN- r, IL-12, IL-18, IL-1β*, and *IL-10* in UPM-IBS/002/2011(IBS002)-infected chicken spleens were significantly upregulated at 3 dpi (Rasoli et al., 2014). However, the immune responses of ducks infected with NDVs of different pathogenicities have not yet been fully explored.

In response to the above mentioned gaps in the literature, in this study, we compared the pathogenicity and mRNA expression of immune-related genes in the lungs and thymuses of ducks at 24, 48, and 72 h post inoculation (hpi) with two current duck-origin epidemic NDV strains of VII genotypes (SS-10) and IX genotypes (NH-10) in China. As a result, we here provide unprecedented evidence regarding host innate immune responses of ducks infected with NDVs.

## Materials and Methods

### Viruses and Animals

The two NDVs used in this study—namely, Duck/CH/GD/SS/10 (SS-10) and Duck/CH/GD/NH/10 (NH-10), were isolated from cloacal swabs of asymptomatic Peking ducks from China’s Guangdong Province during 2010, as previously described (Kang et al., 2014). Healthy 2-week-old Peking ducks were purchased from a duck farm in Guangzhou and housed in the isolator cages. All ducks were confirmed to be serologically negative for ND by hemagglutination inhibition assays for a period of 1 week before experimentation, thereby confirming that the ducks used in this study were clinically healthy. All animal experiments were performed according to an approved animal-use document in animal biosafety level 3 facilities.

### Animal Experiments

Groups (*n* = 14) of healthy 2-week-old Peking ducks were inoculated with 10^6^ of egg infectious dose 50 (EID_50_) of the selected virus via intranasal routes. Three ducks were kept as phosphate-buffered saline-treated and unchallenged controls. At 24, 48, and 72 hpi, three inoculated ducks in each group were euthanized, and the spleen, lung, brain, small intestine, bursa of Fabricius, thymus, and cecal tonsils were collected for the subsequent experiment. The remaining ducks (*n* = 5) were monitored daily for 14 days for clinical symptoms of disease and mortality after challenge. All animal experiments were conducted under the guidance of both of the Centers for Disease Control and Prevention’s Institutional Animal Care and Use Committee and the Association for Assessment and Accreditation of Laboratory Animal Care International. The protocol was approved by the Committee on the Ethics of Animal Experiments of Animal Biosafety Level 3 Committee of South China Agricultural University.

### Virus Titration

The collected tissues (1.0 *g*) were homogenized in 1 mL of phosphate-buffered saline containing ampicillin and penicillin. Debris (2,000 *g*) was pelleted by centrifugation for 5 min, and virus titration was determined in 10-fold serial dilutions of supernatant by inoculation into specific pathogen-free chicken embryos. The virus titers were detected by hemagglutinin testing and calculated using Reed and Muench’s (1938) method.

### *RNA* Extraction and Quantitative Real-time Polymerase Chain Reaction

Total RNA was isolated from the lungs and thymuses using the RNeasy^®^ Mini kit for RNA Purification according to the manufacturer’s instructions (Qiagen Inc., Valencia, CA, USA). RNA was quantified using an Ultrospec 2000 mass spectrophotometer (Pharmacia Biotech, Uppsala, Sweden). Approximately 1 μg of RNA was treated with DNase to remove genomic DNA and used to produce cDNA with a Quantitect^®^ Reverse Transcription kit (Qiagen). Quantitative real-time (qRT) polymerase chain reaction (PCR) was performed in a final volume of 25 μL using 100 ng cDNA, 400 nM of each primer and 12.5 μL QuantiFast SYBR Green PCR Master Mix (Qiagen). Primers used for qRT-PCR (**Table 1**) were designed with Oligo 7 software (Molecular Biology Insights Inc., Cascade, CO, USA) based on target sequences previously reported (Adams et al., 2009). qRT-PCR was performed on an 7500 fast real-time PCR system (Applied Biosystems, Rotkreuz, Switzerland) in a program consisting of one cycle of 95°C for 5 min, followed by 40 cycles of 95°C for 15 s and 60°C for 34°s, after which amplified products of melt-curve analysis were run on a gel and extracted using the QuickClean II DNA gel extraction kit (Qiagen). For the purposes of assay validation, purified products were cloned into pMD19-T and sequenced to verify target amplification.

**Table 1:** Quantitative real-time PCR primers used in this study.

Gene name	Forward primer(5′→3′)	Reverse primer(5′→3′)	GenBank no.
*TLR3*	GAGTTTCACACAGGATGTTTAC	GTGAGATTTGTTCCTTGCAG	JQ910167
*TLR7*	CCTTTCCCAGAGAGCATTCA	TCAAGAAATATCAAGATAATCACATCA	AY940195
*RIG-I*	GCTACCGCCGCTACATCGAG	TGCCAGTCCTGTGTAACCTG	EU363349
*MDA5*	GCTACAGAAGATAGAAGTGTCA	CAGGATCAGATCTGGTTCAG	KF709945
*LGP2*	GTGGTGGAGCTGGAGAAGAG	CCCTGTTCTCCTCAAAGGTG	KC422351
*IL-1*β	TCGACATCAACCAGAAGTGC	GAGCTTGTAGCCCTTGATGC	DQ393268
*IL-2*	GCCAAGAGCTGACCAACTTC	ATCGCCCACACTAAGAGCAT	AF294323
*IL-6*	TTCGACGAGGAGAAATGCTT	CCTTATCGTCGTTGCCAGAT	AB191038
*IL-8*	AAGTTCATCCACCCTAAATC	GCATCAGAATTGAGCTGAGC	DQ393274
*IFN-*a	TCCTCCAACACCTCTTCGAC	GGGCTGTAGGTGTGGTTCTG	EF053034
*IFN-*β	CCTCAACCAGATCCAGCATT	GGATGAGGCTGTGAGAGGAG	AY831397
*IFN-r*	GCTGATGGCAATCCTGTTTT	GGATTTTCAAGCCAGTCAGC	AJ012254
*MHC-I*	GAAGGAAGAGACTTCATTGCCTTGG	CTCTCCTCTCCAGTACGTCCTTCC	AB115246
*MHC-II*	CCACCTTTACCAGCTTCGAG	CCGTTCTTCATCCAGGTGAT	AY905539
*GAPDH*	ATGTTCGTGATGGGTGTGAA	CTGTCTTCGTGTGTGGCTGT	AY436595

### Data Analyses

Each duck cDNA sample was tested in triplicate. Target gene expression was normalized to a constitutively expressed endogenous control gene, glyceraldehyde-3-phosphate-dehydrogenase (GAPDH:AY436595), and the relative gene expression was calculated using the 2^-ΔΔCT^ method (Livak and Schmittgen, 2001). Statistical analyses were performed using GraphPad Prism 6 software (GraphPad Software, Inc., San Diego, CA, USA). NDV virus titer data were compared by using a two-tailed Student’s *t*-test and differences in the expression level of PRRs and pro-inflammatory cytokine genes among ducks infected with NDVs of different pathogenicities were evaluated using two-way ANOVA followed by Duncan’s test. Standard deviations were calculated using the relative expression ratios of three replicates for each gene measured, unless stated otherwise, and results were considered statistically significant when *p*-values were <0.05.

## Results

### Pathogenicity in NDV-Infected Peking Ducks

**FIGURE 1:**
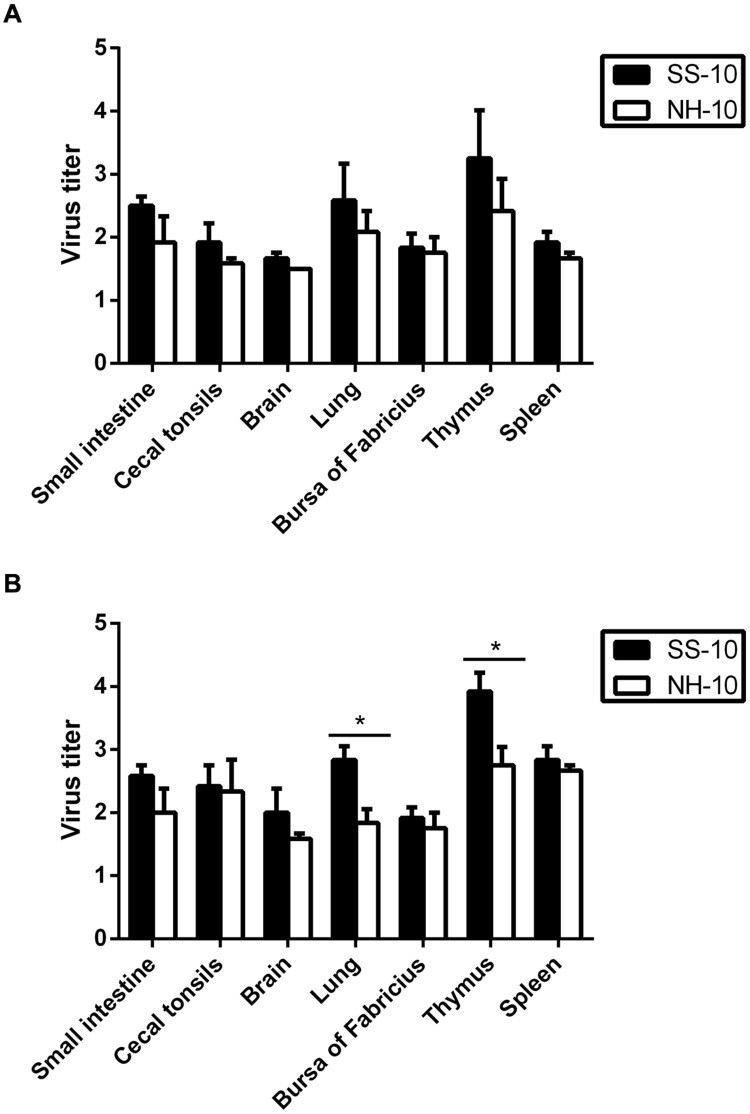
**Replication in Peking ducks of Newcastle disease virus (NDV) after inoculated intranasally.** Healthy 2-week-old Peking ducks were inoculated with 0.2 ml 10^6^ EID_50_ of the selected virus via intranasal routes. Virus titers in Peking ducks at 24 and 48 h post inoculation (hpi). **(A)** 24 hpi; **(B)** 48 hpi. For statistical analysis, a value of 1.5 was assigned if the virus was not detected from the undiluted sample in three embryonated hen eggs. Virus titers are expressed as means ± standard deviation in log_10_ EID_50_/g of tissue. Significance was evaluated with two-tailed Student’s *t*-test (^∗^*p* < 0.05).

In our previous study, we investigated the pathogenicity of the SS-10 and NH-10 viruses in Peking ducks and replicated the viruses in tested tissues of the spleen, lung, brain, small intestine, bursa of Fabricius, thymus, and cecal tonsils with high virus titers at 72 hpi (Kang et al., 2014). To further compare the virulence of these two viruses in Peking ducks, we tested the virus titers in those tissues at 24 and 48 hpi. At 24 hpi, the SS-10 virus systemically replicated in all tested tissues, including those of the small intestine, cecal tonsils, brain, lung, bursa of Fabricius, thymus, and spleen. By contrast, the NH-10 virus was detected in all tested tissues, except that of the brain (**Figure 1A**). The SS-10 and NH-10 viruses replicated highly in the thymus, for mean virus titers of 3.25 and 2.42 log_10_EID_50_, respectively. Both viruses also replicated in the lung for mean titers ranging from 2.08 to 2.58 log_10_EID_50_. At 48 hpi, both viruses caused a systemic infection, and high titers were detected in the thymus of all inoculated ducks (**Figure 1B**), at mean virus titers of 3.92 log_10_EID_50_ and 2.75 log_10_EID_50_, respectively. In the lung, the SS-10 and NH-10 viruses also replicated with mean titer of 2.83 and 1.83 log_10_EID_50_, respectively. At 72 hpi, the virus titers of SS-10 and NH-10 (data not shown) were similar to those of our previous study (Kang et al., 2014) and peaked during the 3 days testing. At no time point was the virus detected in the control groups. Though no ducks inoculated with NH-10 died during the observation period, one of five ducks in the SS-10 group died at 5 dpi. Altogether, our results indicate that SS-10 and NH-10 viruses caused systemic infection in ducks, with high virus titers and replicated quickly in multiple tissues, particularly that of the thymus. They moreover spread to multiple extrapulmonary tissues and exhibited different biological properties in terms of duck pathogenicity.

### Expression of PRRs in the Lung and Thymus Tissues of NDV-Infected Ducks

Of the four known classes of PRR families, Toll-like receptors (*TLR 3/7/8/9*) and *RIG*-*I*-like receptors (i.e., *RIG-I, MDA5, LGP2*) are known to recognize NDV infection (Andrejeva et al., 2004; Lopez et al., 2004; Yoneyama et al., 2004). In this study, we investigated the expression level of PRRs (*TLR3, TLR7, RIG-I, MDA5*, and *LGP2*) in the lungs and thymuses of Peking ducks after NDV infection at 24, 48, and 72 hpi.

**FIGURE 2:**
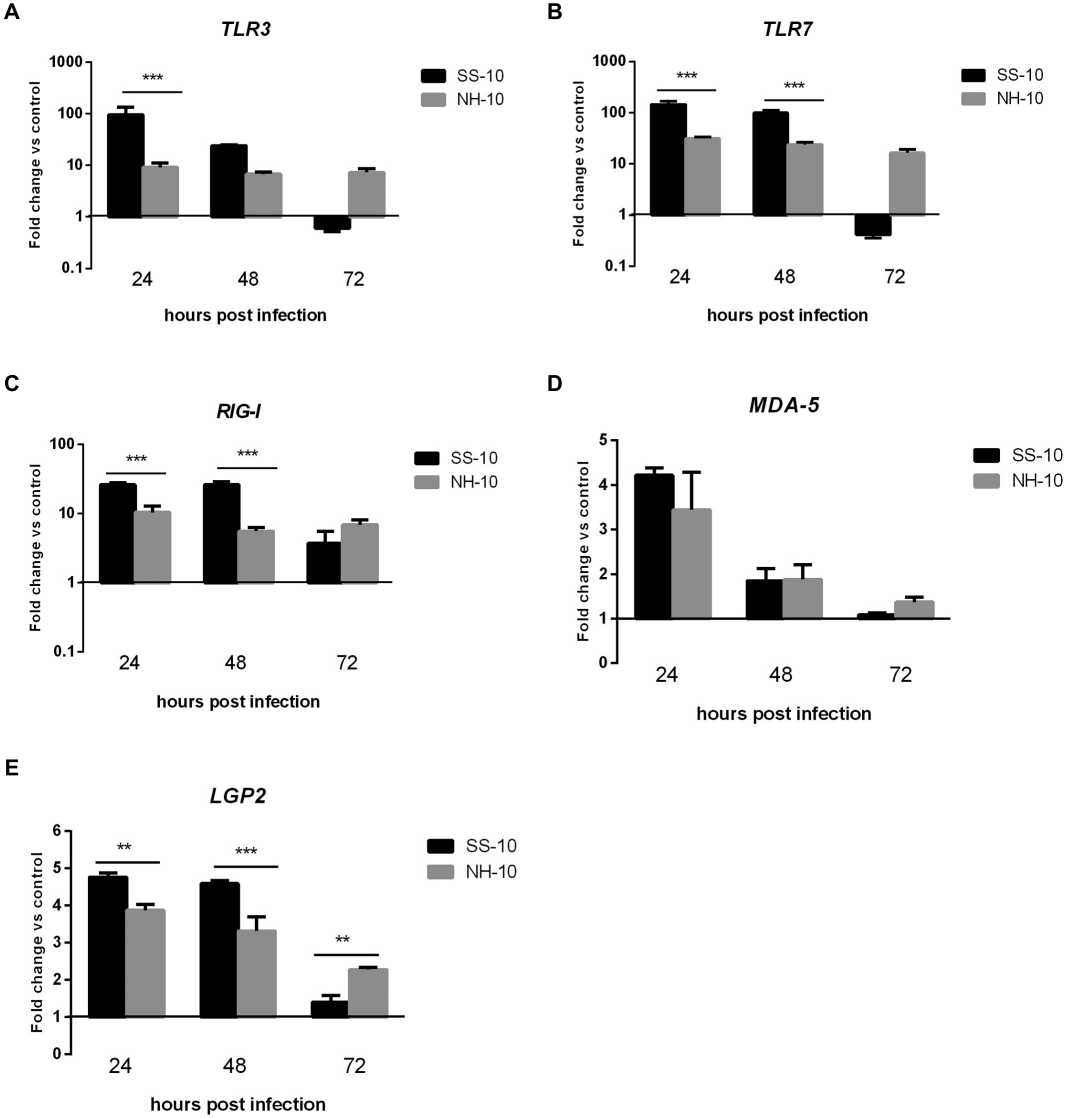
**Fold change expression of pattern recognition receptors (PRRs) in the lungs of infected ducks in response to SS-10 and NH-10. (A)** *TLR3*, **(B)** *TLR7*, **(C)** *RIG-I*, **(D)** *MDA5*, **(E)** *LGP2*. The *Y*-axis represents the fold change in target gene expression in the experimental group relative to those in the control group and presented as the mean values ± standard deviation (*n* = 3). Significance is analyzed with two-way ANOVAs between SS-10 group and NH-10 group at the same time points (^∗^*p* < 0.05, ^∗∗^*p* < 0.01, ^∗∗∗^*p* < 0.001). Error bars indicate standard deviations.

Compared to the mock-infected ducks at 24 hpi, the lungs of SS-10-infected ducks during the first 3 days of infection showed upregulation that peaked in the mRNA expression level of *TLR3* (95.36-fold), *TLR7* (146.35-fold), *RIG-I* (26.29-fold), *MDA5* (4.22-fold), and *LGP2* (4.77-fold), (**Figure 2**). However, the expression of *TLR3* and *TLR7* was downregulated in response to SS-10 infection at 72 hpi (0.61 and 0.41-fold, respectively; **Figures 2A,B**). The lungs of NH-10-infected ducks exhibited expression patterns different from those of SS-10-infected ducks. The expression levels of *TLR3, TLR7*, and *RIG-I* were upregulated during the 3 days of detection, peaked at 24 hpi (9.14-, 31.38-, and 10.42-fold, respectively), and being induced by both viruses, remained high throughout the following 2 days (**Figures 2A–C**). The expression of *MDA5* and *LGP2* was upregulated at 24 hpi (3.44- and 3.87-fold, respectively), which gradually declined to a nadir at 72 hpi (1.37- and 2.28-fold, respectively).

**FIGURE 3:**
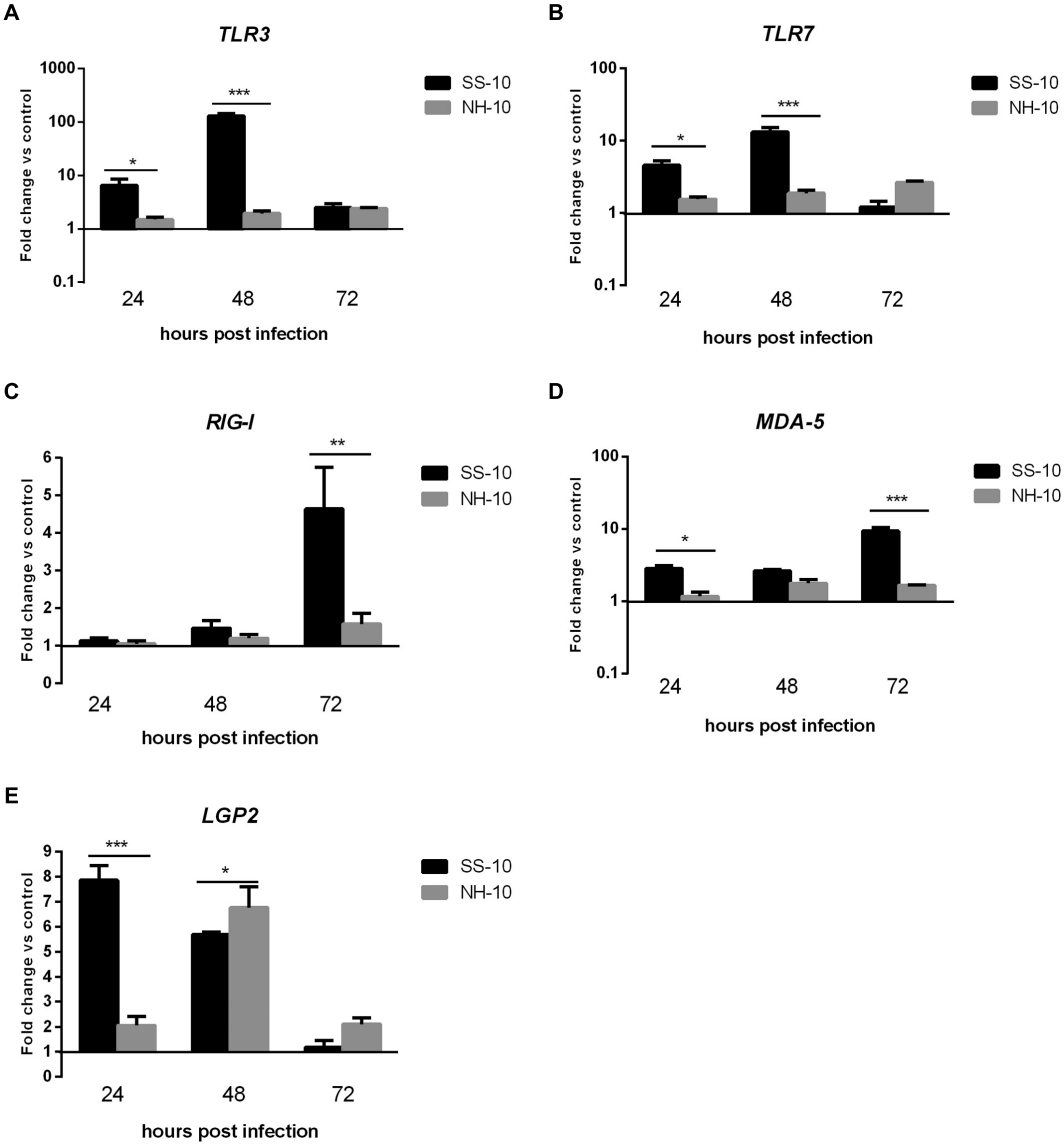
**Fold change expression of PRRs in the thymuses of infected ducks in response to SS-10 and NH-10. (A)** *TLR3*, **(B)** *TLR7*, **(C)** *RIG-I*, **(D)** *MDA5*, **(E)** *LGP2*. The *Y*-axis represents the fold change in target gene expression in the experimental group relative to those in the control group and presented as the mean values ± standard deviation (*n* = 3). Significance is analyzed with two-way ANOVAs between SS-10 group and NH-10 group at the same time points (^∗^*p* < 0.05, ^∗∗^*p* < 0.01, ^∗∗∗^*p* < 0.001). Error bars indicate standard deviations.

In the thymus, by comparison to the NH-10-infected ducks, the expression of *TLR3* and *TLR7* induced by SS-10 was upregulated at 24 hpi (6.51- and 4.55-fold, respectively; *p* < 0.05), peaked at 48 hpi (128.99- and 13.26-fold, respectively; *p* < 0.001), and decreased at 72 hpi (2.51- and 1.21-fold, respectively). By contrast, the expression of *TLR3* and *TLR7* induced by NH-10 was slightly upregulated during 3 days of testing and peaked at 72 hpi (**Figures 3A,B**). The expression of *RIG-I* and *MDA5* induced by both isolated viruses was upregulated during 3 days of detection (**Figures 3C,D**), whereas the expression of *LGP2* was upregulated during the first 3 days of infection and peaked at 24 and 48 hpi, respectively (7.85- and 6.75-fold, respectively; *p* < 0.05). In general, the two viruses induced a significant upregulation of PRRs in the lung and thymus tissues of infected ducks, though SS-10-infected lungs and thymus exhibited greater upregulation than NH-10-infected lungs and thymuses.

### Expression of Cytokine and Chemokine in Lung and Thymus Tissues of NDV-Infected Ducks

**FIGURE 4:**
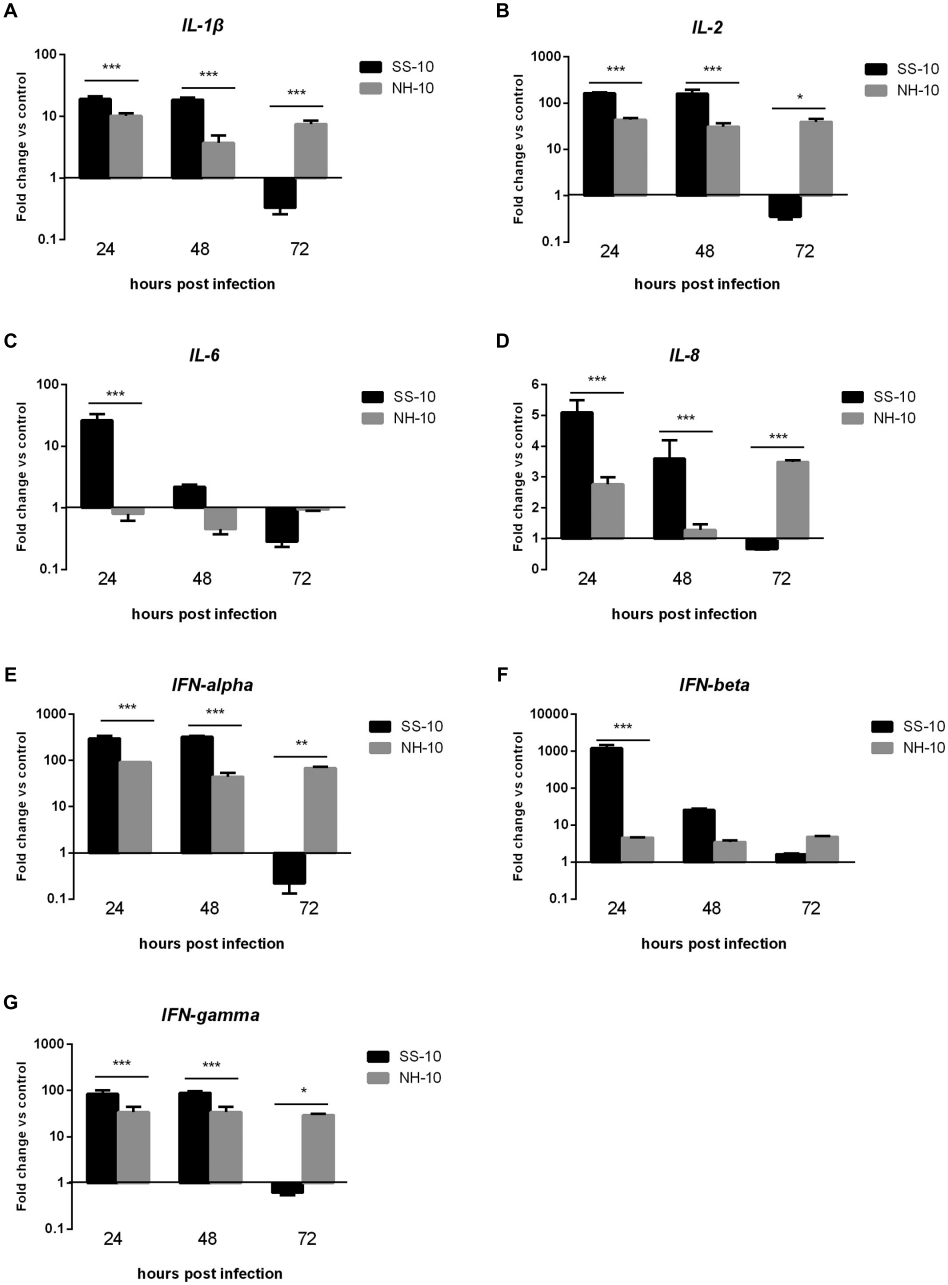
**Fold change expression of cytokines in the lungs of infected ducks in response to SS-10 and NH-10. (A)** *IL-1*β, **(B)** *IL-2*, **(C)** *IL-6*, **(D)** *IL-8*, **(E)** *IFN-alpha*, **(F)** *IFN-beta*, **(G)** *IFN-gamma*. The *Y*-axis represents the fold change in target gene expression in the experimental group relative to those in the control group and presented as the mean values ± standard deviation (*n* = 3). Significance is analyzed with two-way ANOVAs between SS-10 group and NH-10 group at the same time points (^∗^*p* < 0.05, ^∗∗^*p* < 0.01, ^∗∗∗^*p* < 0.001). Error bars indicate standard deviations.

Pro-inflammatory cytokine and chemokine are primarily mediators of the host innate immune response during virus infection and are potentially involved in antimicrobial defense (Takeuchi and Akira, 2010). To compare cytokine responses in ducks infected with SS-10 and NH-10, the expression of pro-inflammatory cytokines (*IL-1*β, *IL-6*), chemokine (*IL-8*), Th1-involved cytokine (*IL-2*), and IFN types I and II (*IFN-alpha, IFN-beta*, and *IFN-gamma*) in the lungs and thymus of ducks were tested during early-phase post-infection. In the lungs, the expression level of *IL-1*β induced by SS-10 was upregulated by 19.14- and 18.69-fold at 24 and 48 hpi, yet downregulated at 72 hpi (0.33-fold); however, induced by NH-10, the expression of *IL-1*β was upregulated throughout the duration of the experiment (**Figure 4A**). The expression level of *IL-6* in SS-10-infected ducks was upregulated at 24 and 48 hpi, but suppressed at 72 hpi. By contrast, the expression of *IL-6* was down-regulated when induced by NH-10 during 3 days of testing (**Figure 4C**). Furthermore, the expression levels of *IL-2* and *IL-8* showed a similar pattern of increase, with upregulation by 30.98- to 163.73-fold and 1.28- to 5.09-fold at 24 and 48 hpi, respectively, though not at 72 hpi (**Figures 4B,D**). The expression levels of IFN types I and II (i.e., *IFN-alpha, IFN-beta*, and *IFN-gamma*) were significantly greater in the lungs of SS-10-infected ducks at 24 hpi (325.13-, 1216.58- and 88.57-fold, respectively; *p* < 0.001) than NH-10-infected ducks (**Figures 4E,F**). Notably, *IFN-beta* was greatly induced by SS-10 and markedly upregulated at 24 hpi (1216.58-fold; *p* < 0.001) when compared to NH-10-infected ducks.

**FIGURE 5:**
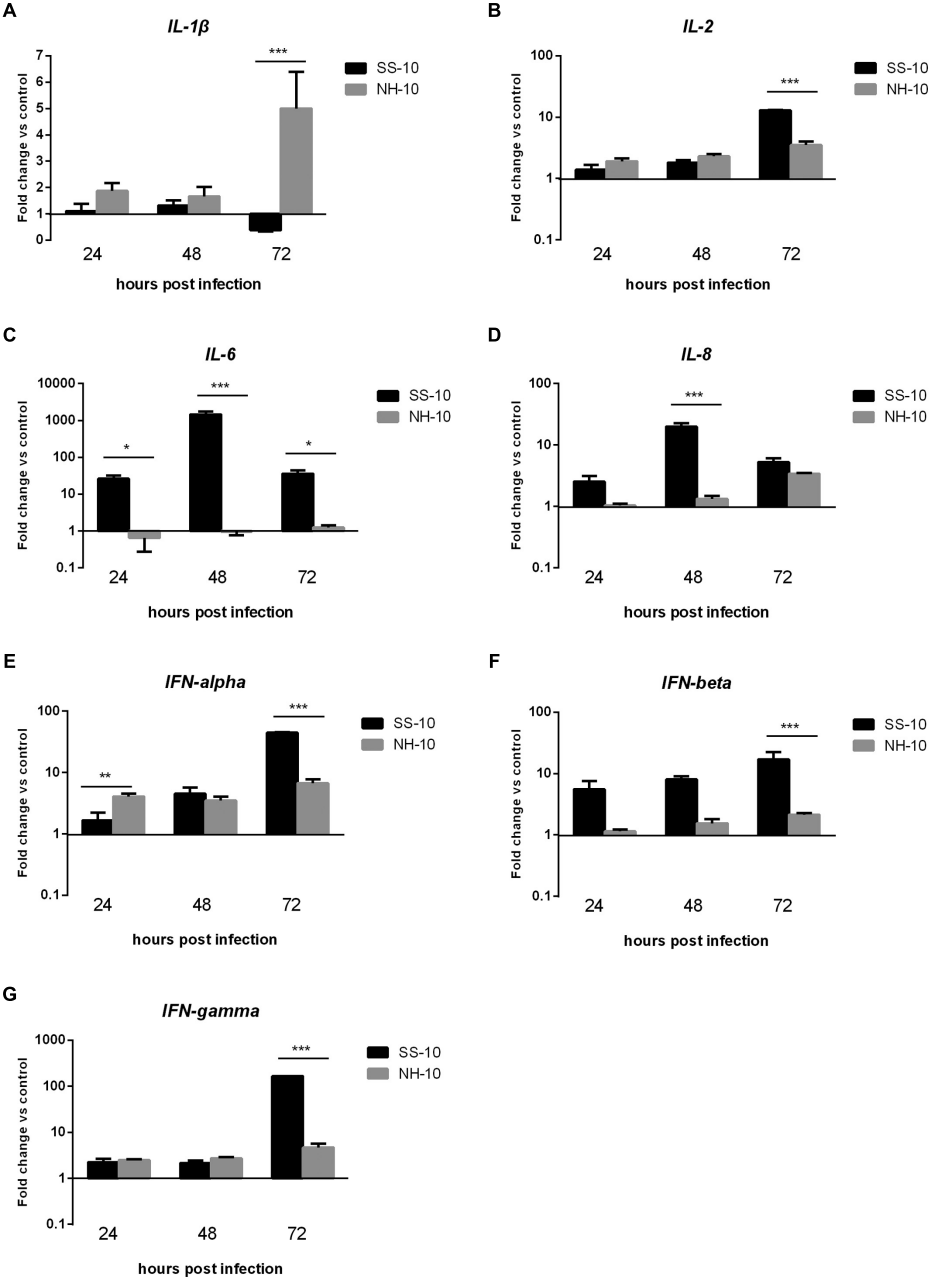
**Fold change expression of cytokines in the thymuses of infected ducks in response to SS-10 and NH-10. (A)** *IL-1*β, **(B)** *IL-2*, **(C)** *IL-6*, **(D)** *IL-8*, **(E)** *IFN-alpha*, **(F)** *IFN-beta*, **(G)** *IFN-gamma*. The *Y*-axis represents the fold change in target gene expression in the experimental group relative to those in the control group and presented as the mean values ± standard deviation (*n* = 3). Significance is analyzed with two-way ANOVAs between SS-10 group and NH-10 group at the same time points (^∗^*p* < 0.05, ^∗∗^*p* < 0.01, ^∗∗∗^*p* < 0.001). Error bars indicate standard deviations.

In the thymus, the expression level of pro-inflammatory cytokine *IL-1*β was slightly upregulated compared to the uninfected control following SS-10 or NH-10 infection at 24 and 48 hpi. Though its expression was downregulated in response to infection with SS-10 in ducks at 72 hpi, its expression increased in response to infection with NH-10 in ducks at 72 hpi compared to the negative control (**Figure 5A**). Induced by NH-10, *IL-6* expression was downregulated at 24 and 48 hpi (0.65- and 0.96-fold, respectively) and later upregulated at 72 hpi (1.25-fold); however, it was upregulated on the following test day and peaked at 1,454.69-fold expression at 48 hpi when induced by SS-10 (**Figure 5C**). The expression level of *IL-2* was upregulated at all time points when induced by SS-10 and NH-10, and peaked at 72 hpi (12.89- and 3.52-fold, respectively). The expression level of *IL-8* was upregulated when induced by both viruses for the duration of the experiment, yet was greater for SS-10 than NH-10 (**Figure 5D**). Furthermore, *IFN-alpha, IFN-beta*, and *IFN-gamma* showed similar tendencies of increased expression when induced by both viruses during the tested periods, eventually peaking at 72 hpi, though greater for SS-10 than for NH-10 at 72 hpi (**Figures 5E,F**).

These results indicate that SS-10 induced the mRNA expression level of cytokines and chemokine, such as *IL-1*β, *IL-2, IL-6, IL-8, IFN-alpha, IFN-beta*, and *IFN-gamma* to a greater extent than NH-10 in the lungs and thymuses of infected ducks, suggesting that they play important roles in resisting NDV infection. In sum, the expression of various cytokines and chemokines are involved in the host innate immune response to NDV, though the expression patterns of some cytokines can vary.

### Expression of Major Histocompatibility Complex (MHC) Classes I and II in the Lung and Thymus Tissues of NDV-Infected Ducks

**FIGURE 6:**
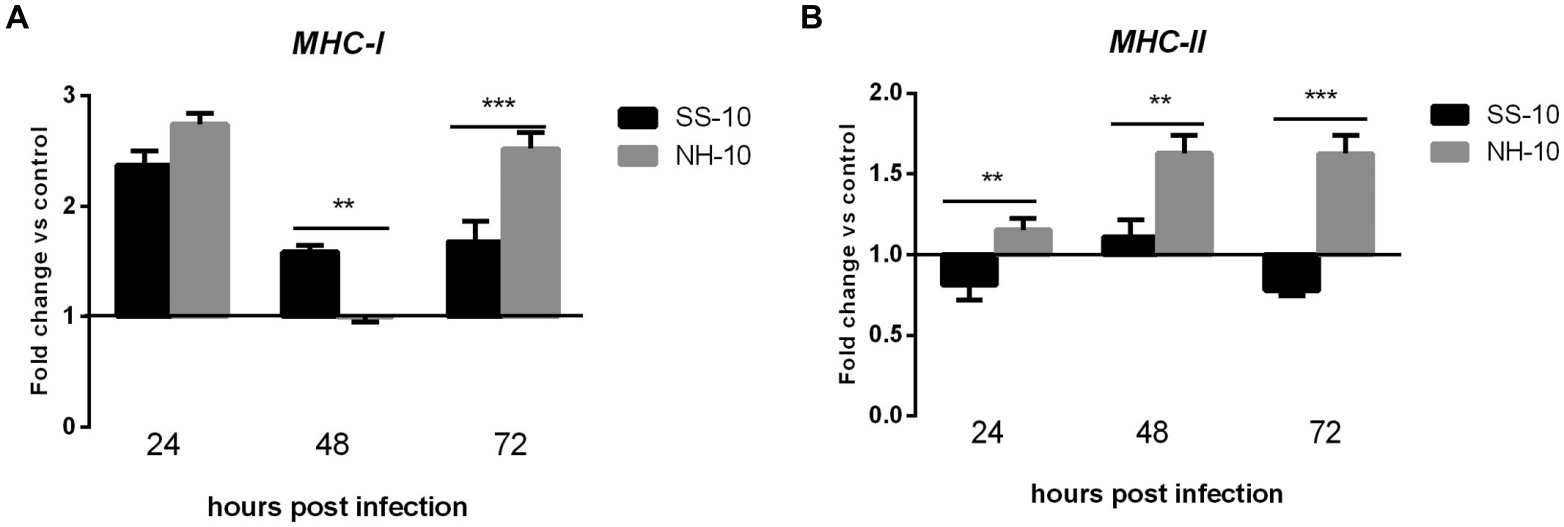
**Fold change expression of MHC in the lungs of infected ducks in response to SS-10 and NH-10. (A)** *MHC-I*, **(B)** *MHC-II*. The *Y*-axis represents the fold change in target gene expression in the experimental group relative to those in the control group and presented as the mean values ± standard deviation (*n* = 3). Significance is analyzed with two-way ANOVAs between SS-10 group and NH-10 group at the same time points (^∗^*p* < 0.05, ^∗∗^*p* < 0.01, ^∗∗∗^*p* < 0.001). Error bars indicate standard deviations.

We next investigated whether the mRNA expression level of *MHC-I* and *MHC-II*, typically regulated after NDV infection, correlated with early-phase host innate immune response in ducks. *MHC-I* expression level was upregulated in the lungs of infected ducks when induced by both viruses throughout the duration of the experiment. Specifically, the expression level of *MHC-I* was upregulated when induced by both viruses at 24 hpi (2.74- and 2.37-fold, respectively) compared to uninfected controls, decreased slightly, plateaued at the baseline with no significant changes at 48 hpi (1.59- and 1.00-fold, respectively), and then slightly increased at 72 hpi (2.52- and1.68-fold, respectively; **Figure 6A**). The expression level of *MHC-II* was slightly upregulated throughout the experimental period when induced by NH-10; however, the mRNA expression level of *MHC-II* in SS-10-infected ducks was downregulated at 24 hpi (0.81-fold), and upregulated at 48 hpi (1.11-fold), yet again downregulated at 72 hpi (0.78-fold; **Figure 6B**).

**FIGURE 7:**
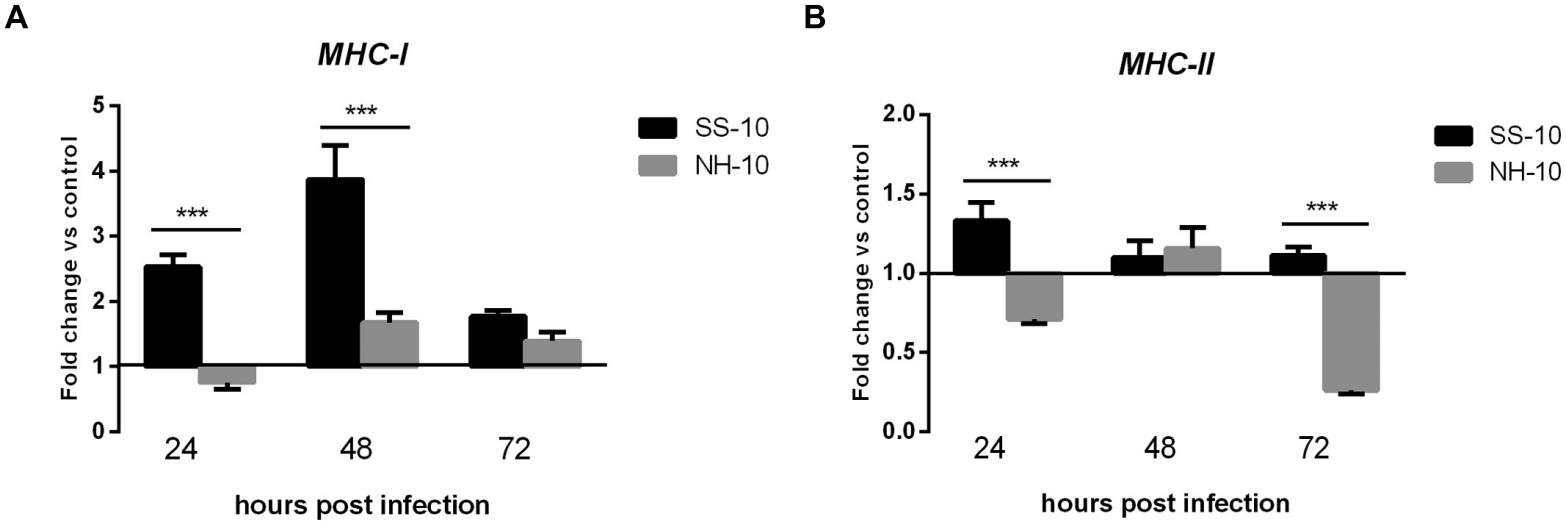
**Fold change expression of MHC in the thymuses of infected ducks in response to SS-10 and NH-10. (A)** *MHC-I*, **(B)** *MHC-II*. The *Y*-axis represents the fold change in target gene expression in the experimental group relative to those in the control group and presented as the mean values ± standard deviation (*n* = 3). Significance is analyzed with two-way ANOVAs between SS-10 group and NH-10 group at the same time points (^∗^*p* < 0.05, ^∗∗^*p* < 0.01, ^∗∗∗^*p* < 0.001). Error bars indicate standard deviations.

The thymuses of NH-10-infected ducks showed expression patterns different from those of SS-10-infected ones. The expression levels of *MHC-I* and *MHC-II* were upregulated when induced by SS-10 during 3 days of observation (**Figures 7A,B**). However, the expression level of *MHC-I* induced by NH-10 was downregulated at 24 hpi (0.76-fold), slightly upregulated at 48 hpi (1.68-fold), but decreased somewhat at 72 hpi (1.39-fold) compared to the uninfected control (**Figure 7A**). Additionally, the expression level of *MHC-II* induced by NH-10 was downregulated at 24 hpi, slightly upregulated at 48 hpi (1.16-fold), yet again downregulated at 72 hpi (**Figure 7B**). In all, these data indicate that the expression levels of *MHC-I* and *MHC-II* induced by NDV infection are at least partially involved in host innate immune responses in ducks.

## Discussion

Since NDV’s initial isolation in China in 1948 and following a strict vaccination program and the culling of infected poultry, the sporadic occurrences of ND have been mild and caused few deaths in domestic poultry. However, field outbreaks of ND have continually occurred in the vaccinated poultry of China since 1996 (Liu et al., 2003; Jinding et al., 2005). NDV’s varied pathogenicity is a complex trait determined by multiple genetic factors and primarily depends on the consequences of virulence and host innate immune response. Several studies have illustrated that immune-related cytokine genes are regulated in host innate immune response to NDV infection in chickens peripheral blood and spleen tissue (Rue et al., 2011; Hu et al., 2012; Liu et al., 2012). In 2010, two duck-origin NDV strains, SS-10 and NH-10, were genotypically and pathotypically characterized (Kang et al., 2014). In the present study, we compared the pathogenicity and mRNA expression of immune-related genes in ducks in response to two predominant duck-origin genotypes circulating in South China.

The pathogenicity of NDV in ducks correlates directly with the virulence of the virus in tissues. In this study, SS-10 and NH-10 caused systemic infection, especially in the primary target tissues of virulent NDV strains such as those of the spleen, bursa of Fabricius, thymus, and cecal tonsils, but not the brain, when induced by NH-10, and the highest titers of virus were detected in the thymus of all inoculated ducks during the period of infection. Furthermore, these two strains viral titer at 24 hpi were greatest in the lung, though not as great as in the thymus, indicating that both viruses replicate rapidly in the parenchymal tissues, including those of the lung (**Figure 1**). These results demonstrate that these two NDV strains infection affects almost all vital tissues of ducks, and induced severe respiratory and gastrointestinal dysfunctions, as well as hemorrhagic lesions, and spread to multiple extrapulmonary tissues and exhibited different pathogenicity in ducks.

Newcastle disease virus infection is recognized by host PRRs, such as *TLR3/7/8, RIG-I, MDA-5*, and *LGP2*, induces both antiviral responses, including IFN type I production, and the subsequent eradication of replicating viral genomes (Yoneyama et al., 2004). A previous study has demonstrated that *RIG-I* is significantly upregulated and reached the highest levels in the lungs, air sacs, and embryo fibroblast cells of geese at 1 dpi, yet generally decreased at 3 dpi, when infected with NDV (Sun et al., 2013). Moreover, *TLR7’*s mRNA expression levels were upregulated in the spleens of both AF2240- and IBS002- infected chickens at 1, 3, and 4 dpi (Rasoli et al., 2014). However, *TLR3’*s mRNA expression level was downregulated at 1 dpi, upregulated at 3 dpi, and downregulated again at 4 dpi in IBS002-infected chicken spleens (Rasoli et al., 2014). In the current study, *TLR3, TLR7, RIG-I*, and *MDA5* genes were significantly upregulated in the lungs and thymuses of SS-10- and NH-10-infected ducks compared to the mock-infected ducks at several time points, although the expression of *TLR3* and *TLR7* was downregulated in the lungs when induced by SS-10 at 72 hpi (**Figures 2** and **3**). Additionally, the expression level of *LGP2* was upregulated in the lungs and thymuses when induced by SS-10 and NH-10 for during the 3 days of observation (**Figures 2E** and **3E**), as generally consistent with earlier research (Komuro and Horvath, 2006). Therefore, Toll-like and RIG-I-like receptors are involved in the early-phase host innate immune response of ducks to SS-10 and NH-10 infection, yet demonstrated varied pathogenicity in these birds.

The involvement of pro-inflammatory cytokines and chemokines in antiviral host innate immune responses depends on the recognition of viral components by host pattern-recognition receptors (Kawai and Akira, 2006). Research has shown that infected chicken cells and tissues express pro-inflammatory cytokines and chemokines during early-phase response to NDV infection (Rue et al., 2011; Hu et al., 2012; Kapczynski et al., 2013; Sun et al., 2013; Rasoli et al., 2014). The cytokines *IFN-alpha, IFN-gamma, IL-1*β and *IL-6* became significantly induced following infection with NDV-CA02 in chicken splenocytes *in vitro* during early-phase post-infection (Rue et al., 2011). In the present study, we also observed the upregulation of *IL-1*β, *IL-8, IFN-alpha*, and *IFN-gamma* in the lungs and thymuses of SS-10-infected ducks at 24 and 48 hpi (**Figures 4** and **5**). However, the expression levels of *IL-1*β, *IL-2, IL-8, IFN-alpha*, and *IFN-gamma* were downregulated in the lungs following SS-10 infection at 72 hpi (**Figure 4**). Interestingly, in this study the expression level of *IL-6* was downregulated in the lungs and thymuses in response to NH-10 infection throughout the duration of the experiment (**Figures 4C** and **5C**). Yet, the expression level of *IL-6* was upregulated in the lungs and thymuses of SS-10-infected ducks during the 3 days of observation, though not in the lungs at 72 hpi (**Figures 4C** and **5C**). Most importantly, the expression level of *IL-6* was remarkably upregulated (1,454.69-fold, *p* < 0.05) in the thymus when induced by SS-10 at 48 hpi. Consistent with these observations, we have shown here that *IFN-beta* was upregulated to a remarkably high level (1,216.58-fold, *P* < 0.001) in the lungs after infection with SS-10 compared to NH-10-infected ducks at 24 hpi. At the same time, the low pathogenic NH-10 strain induced a far lesser expression of *IFN-beta* genes in the lung and thymus, chiefly due to the antagonism of the IFN response by NDV V protein, thereby granting the virus a period of stealth replication. The viral subversion of IFN by a low pathogenic NDV strain was observed in avian cells, while a V truncation mutant was a strong IFN inducer (Huang et al., 2003). In mammals, cytokine storms, characterized by a dys-regulated and exaggerated production of inflammatory cytokines in turn correlated with increased morbidity and mortality during multiple pathogenic respiratory viral infections (Kobasa et al., 2007; MacNeil et al., 2011). In the present study, SS-10 caused one of the five inoculated ducks to die at 5 dpi, though no ducks from the NH-10 groups died during the 14-d observation period. These results demonstrate that the response of overabundant cytokines, such as *IL-6* and *IFN-beta* to NDV infection is not critical for the efficient elimination of the virus and insufficient for protecting duck hosts from death. On the contrary, it is more likely the direct result of an increased viral burden as a consequence of enhanced virulence that in turn results in severe immunopathological damage, if not death as well.

The consequences of the up- or downregulation of *MHC-I* and *MHC-II* in NDV-infected duck tissues to both the virus and host remain unclear. A previous study reported that the expression level of *MHC-I* induced by NDV-CA02 was downregulated in spleens at 1 dpi and upregulated at 2 dpi (Hu et al., 2012). Moreover, the expression level of *MHC-II* induced by NDV-AF2240 was downregulated throughout the infection period (Rasoli et al., 2014). In agreement with these studies, we have shown that the *MHC-I* and *MHC-II* genes were upregulated in thymus tissue following SS-10 infection after the 3 days of testing (**Figures 7A,B**). However, the *MHC-II* gene was downregulated in the lungs when induced by SS-10 at 24 hpi, upregulated at 48 hpi, and downregulated again at 72 hpi (**Figure 6A**). Conversely, the expression levels of *MHC-I* and *MHC-II* in the lungs and thymus induced by NH-10 showed a different pattern; they increased slightly in the lungs of ducks post-infection with the NH-10 virus (**Figures 6A,B**). As such, these findings suggest that NDV has a varying ability to modulate the mRNA expression of *MHC-I* and *MHC-II*.

## Conclusion

We demonstrated the host innate immune-related gene expression patterns of ducks associated with different pathogenicities of NDV infection. The expression profile is remarkable both given its rapid timing and the magnitude of gene induction in the lungs and thymus following NDV infection. These findings thereby contribute to a comprehensive comparison of innate, intrinsic immunity against NDV in duck host innate immune response.

## Conflict of Interest Statement

The authors declare that the research was conducted in the absence of any commercial or financial relationships that could be construed as a potential conflict of interest.
